# Comparative Omics Analysis of *Brassica napus* Roots Subjected to Six Individual Macronutrient Deprivations Reveals Deficiency-Specific Genes and Metabolomic Profiles

**DOI:** 10.3390/ijms222111679

**Published:** 2021-10-28

**Authors:** Galatéa Courbet, Aurélien D’Oria, Anne Maillard, Lun Jing, Sylvain Pluchon, Mustapha Arkoun, Stéphanie Pateyron, Christine Paysant Le Roux, Sylvain Diquélou, Alain Ourry, Jacques Trouverie, Philippe Etienne

**Affiliations:** 1 Unicaen, Inrae, UMR 950 Eva, SFR Normandie Végétal (FED4277), Normandie Université, 14000 Caen, France; galatea.courbet@unicaen.fr (G.C.); aurelien.doria@unicaen.fr (A.D.); sylvain.diquelou@unicaen.fr (S.D.); alain.ourry@unicaen.fr (A.O.); jacques.trouverie@unicaen.fr (J.T.); 2 Laboratoire de Nutrition Végétale, Agro Innovation International-TIMAC AGRO, 35400 Saint-Malo, France; anne.maillard@roullier.com (A.M.); sylvain.pluchon@roullier.com (S.P.); mustapha.arkoun@roullier.com (M.A.); 3 Plateformes Analytiques de Recherche, Agro Innovation International-TIMAC AGRO, 35400 Saint-Malo, France; lun.jing@roullier.com; 4 Institute of Plant Sciences Paris-Saclay (IPS2), Université Paris-Saclay, CNRS, INRAE, Univ Evry, 91405 Orsay, France; stephanie.pateyron@inrae.fr (S.P.); christine.paysant-le-roux@inrae.fr (C.P.L.R.)

**Keywords:** macronutrient deprivations, metabolic pathways, metabolomic, molecular indicators, regulations of nutrient metabolism, transcriptomic

## Abstract

The early and specific diagnosis of a macronutrient deficiency is challenging when seeking to better manage fertilizer inputs in the context of sustainable agriculture. Consequently, this study explored the potential for transcriptomic and metabolomic analysis of *Brassica napus* roots to characterize the effects of six individual macronutrient deprivations (N, Mg, P, S, K, and Ca). Our results showed that before any visual phenotypic response, all macronutrient deprivations led to a large modulation of the transcriptome and metabolome involved in various metabolic pathways, and some were common to all macronutrient deprivations. Significantly, comparative transcriptomic analysis allowed the definition of a subset of 3282, 2011, 6325, 1384, 439, and 5157 differentially expressed genes (DEGs) specific to N, Mg, P, S, K, and Ca deprivations, respectively. Surprisingly, gene ontology term enrichment analysis performed on this subset of specific DEGs highlighted biological processes that are common to a number of these macronutrient deprivations, illustrating the complexity of nutrient interactions. In addition, a set of 38 biochemical compounds that discriminated the macronutrient deprivations was identified using a metabolic approach. The opportunity to use these specific DEGs and/or biochemical compounds as potential molecular indicators to diagnose macronutrient deficiency is discussed.

## 1. Introduction

Nitrogen (N), magnesium (Mg), phosphorus (P), sulfur (S), potassium (K), and calcium (Ca) are macronutrients present in large amounts in plant tissues (more than 0.1% of dry weight) and are essential for plant growth, seed yield, and nutritional quality [1,2]. In higher plants, severe macronutrient deficiencies are usually associated with phenotypic symptoms [3] such as leaf chlorosis [4], root ramification [5], and, ultimately, reduction in plant growth [6]. In addition, a single macronutrient deficiency commonly leads to profound alterations in the elemental content of plant tissues, reflecting a modulation of the uptake of several nutrients with a corresponding modification of plant ionomic composition [7,8,9,10]. While the root uptake of other nutrients is reduced by individual deficiencies in most cases, there are also many instances of increased mineral uptake that result from the numerous crosstalks between nutrients. For example, S deficiency [9,10,11] is associated with an increase in plant contents of molybdenum (Mo), selenium (Se), and vanadium (V) because root sulfate transporters, which are up-regulated under S deficiency, are also able to mediate transport of these structurally similar anions. Contrastingly, one macronutrient deficiency can massively decrease the uptake of another. This can be illustrated by N deficiency, which massively decreases sodium (Na) uptake [9].

Since macronutrients are involved in many crucial metabolic pathways [12,13], all macronutrient deficiencies have a myriad of effects on plant metabolism [3]. For example, because N is incorporated into organic compounds such as amino acids and nucleic acids, a limitation of this element leads to a defect in protein synthesis that affects various metabolic pathways [14,15]. Similarly, as S is a constituent of two amino acids, cysteine and methionine, S deprivation also disrupts many metabolic pathways, including nitrogen metabolism [16,17]. Moreover, due to the requirement for S in the synthesis of molecules such as glutathione and S adenosine methionine (ethylene precursor), its depletion leads to the alteration of pathways involved in the mitigation of biotic and abiotic stresses [18]. Likewise, Mg and P are important components of biomolecules, with Mg essential for chlorophylls, and P being a key component of adenosine triphosphate (ATP), nucleic acids, and phospholipids [19,20], so they are both crucial for plant cell functioning. The central structural role that Ca plays in cell wall and plasma membrane organization means that Ca deficiency results in cell death and growth cessation [3,21]. More broadly, Ca is an ubiquitous and versatile second messenger in plants [22], and Ca deprivation alters a wide range of plant metabolic pathways [3,23]. Finally, although K is mainly involved in cellular osmoregulation and in the maintenance of cation–anion balances, it is well documented in several plants species that K deficiency negatively affects photosynthesis processes in particular, and mostly via alterations of stomatal movement, enzyme activation, and protein synthesis and through overproduction of reactive oxygen species (ROS) [24,25,26].

Over the past few decades, access to the genomes of numerous species has favored the emergence of transcriptomic approaches that allow a more integrated view of plant responses to different nutritional deprivations. Thus, a range of studies across diverse plant species have used transcriptomic approaches to decipher metabolic changes in response to a given macronutrient deprivation. For example, in rice deprived of N for 12 h, it was observed that among the 1650 differentially expressed genes (DEGs), more than 30% were classified into the Gene Ontology (GO) categories “metabolic process”, “stimulus response”, and “biological regulation” [27]. In another study [28], authors reported that durum wheat responded to a long-time N starvation (until the late milk developmental stage Z77) by modulating the expression of 4626 genes mainly classified into the following GO categories: N compound metabolism, C metabolism, photosynthesis, N transport, and N assimilation. Similar approaches focusing on S deficiency have shown that among the 632 DEGs in S-deprived plants, most were related to the sulfur assimilation pathway but also to the flavonoid, auxin, and jasmonate biosynthetic pathways [29]. Other authors have demonstrated that variations in sulfur availability modulates the expression of genes involved in the regulation of S, N, and P metabolisms [30,31,32], highlighting the strong interactions between S and other elements. Previously, it has been reported that K deficiency modulates the expression of a smaller panel of genes than N or P deficiencies [33]. In many plant species, several studies have shown that the majority of DEGs identified in K-starved plants are linked to phytohormones (especially auxin and jasmonate), cation transporters (especially iron, zinc, and Ca transporters), Ca signaling, and protein phosphorylation [33,34,35,36,37]. Concerning transcriptomic analysis of P-starved plants, a study performed on wheat identified several DEGs associated with plant defense, plant stress responses, nutrient mobilization, or pathways involved in the gathering and recycling of phosphorus [38]. In another study authors reported that among the 1644 DEGs in roots of P-deprived plants, most of them belonged to pathways involving phosphorylated metabolites such as nucleic acids and phospholipids [39]. Nevertheless, P is not the only element able to modulate phosphorylation pathways. Indeed, it was previously demonstrated that *Citrus sinensis* adapts to Mg privation in association with DEGs implicated in cell cycle regulation, signal transduction, photosynthesis, cell wall remodeling, and the antioxidant system, as well as phosphorylation control [40]. Finally, among all the macronutrients, Ca deprivation is the least studied. However, a large set of genes are known to be regulated in Ca-starved rice plants (5588 DEGs after 14 days) and their functional classification allocated them to processes related to growth and development such as ion transport, signal transduction, and transcriptional regulation [41].

These large-scale studies have provided valuable data to the scientific community exploring macronutrient deficiencies. Nevertheless, due to a diversity in the genotypes used and the growing conditions, treatment durations, and intensity of the limitations applied, it is difficult to formulate an integrative and comparative overview of the responses of a given plant species to all individual macronutrient deprivations. In this study, we propose to overcome this difficulty by performing simultaneous deprivation of each macronutrient (N, P, K, S, Mg, or Ca) in *Brassica napus* plants cultivated in controlled conditions. The experiment was designed so that plants were subjected to total deprivation but harvested before any significant effect on plant growth in order to detect the earliest processes affected by macronutrient deficiencies. The first aim of this study was to characterize the molecular response for each macronutrient deficiency by using transcriptomic and metabolomic analyses in roots, the first tissues that experience a nutrient deficiency. Based on these global data and using comparative analyses, the second aim was to define DEG lists and metabolomic profiles specific to each macronutrient deprivation. Because accurate molecular indicators of single macronutrient deprivations are lacking for *Brassica napus*, we discuss the opportunity to use these specific gene and/or metabolite sets for early diagnosis of each macronutrient deprivation before manifestation of any growth alteration and/or visual symptoms.

## 2. Results

### 2.1. A 10-Day Single Macronutrient Deprivation Had Minor Effects on Biomass and Photosynthetic Activity

Compared to the control, the root and shoot biomasses of rapeseed plants subjected to single macronutrient deprivations for 10 days were not significantly altered, with the exception of the root biomass of P-deficient plants, which increased significantly by about 30% (Table 1). Nevertheless, the –P shoot/root ratio was not significantly altered (5.0 ± 0.4 and 4.5 ± 0.5 for P-deprived and control plants, respectively), which was similar to all the other macronutrient deprivations.

Regardless of the treatments (control and macronutrient deprivation), leaf photosynthetic activities were similar until day 6 (Supplemental Data SD8). At day 9, only N and P deprivations resulted in a significant decrease in photosynthetic activity compared to the control of about 62% and 30%, respectively (Table 1). To check the effect of deprivation, total net uptake of each macronutrient was evaluated per plant (control or deprived) as the difference between plant content before and after 10 days of deficiency. For most macronutrients, plant uptake was marginal during the 10 days of deficiency, except for N, whose uptake was only reduced by 73% compared to control plants (Table 1). This may be easily explained by plant heterogeneity (at D0 and/or at 10 days) rather than by of nutrients that may have been left in the deprived nutrient solution.

### 2.2. Overview of the Root Transcriptome of Brassica napus Deprived of N, Mg, P, S, K, or Ca for 10 Days

**Figure 1:**
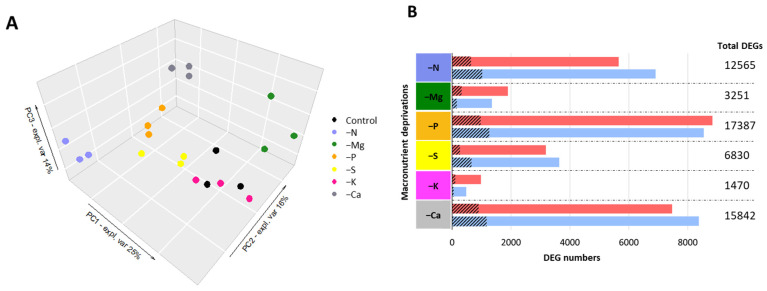
(**A**) Principal component analysis (PCA) score plots of normalized counts of *Brassica napus* roots projected onto the subspace spanned by components 1 (PC 1), 2 (PC2), and 3 (PC3). Roots samples (*n* = 3) are colored according to the nutritional status (control and macronutrient deprived). (**B**) Number of total DEGs and upregulated (red) and downregulated (blue) differentially expressed genes (DEGs). Hatched areas correspond to the number of DEGs with “unknown” annotation.

Principal component analysis (PCA) of the normalized RNA-seq counts showed that replicates for each condition (control and macronutrient deprivations) are clustered. Except for control and K deprivation, clusters segregated from each other along the three first principal component axes, which explained 25%, 16%, and 14% of the overall variability of the root samples, respectively (Figure 1A).

The count of total DEGs (up- and down- regulated compared to the control; *p* ≤ 0.05) for each macronutrient deprivation was highly variable (Figure 1B). Thus, P, Ca, and N deprivations affected the transcriptome the most with 17,387 (8839 upregulated and 8548 downregulated), 15,842 (7469 upregulated and 8373 downregulated), and 12,565 (5653 upregulated and 6912 downregulated) DEGs, respectively. For example, deprivation of S and Mg revealed about two- and fourfold fewer DEGs (6830 and 3251 DEGs, respectively) than N deprivation. Finally, K deprivation showed comparatively restricted effects on the transcriptome, with a total of only 1470 DEGs, of which 984 and 486 were up- and down- regulated, respectively (Figure 1B).

### 2.3. Functional Classification of DEGs for Each Macronutrient Deprivation in Roots of Brassica napus

**Figure 2:**
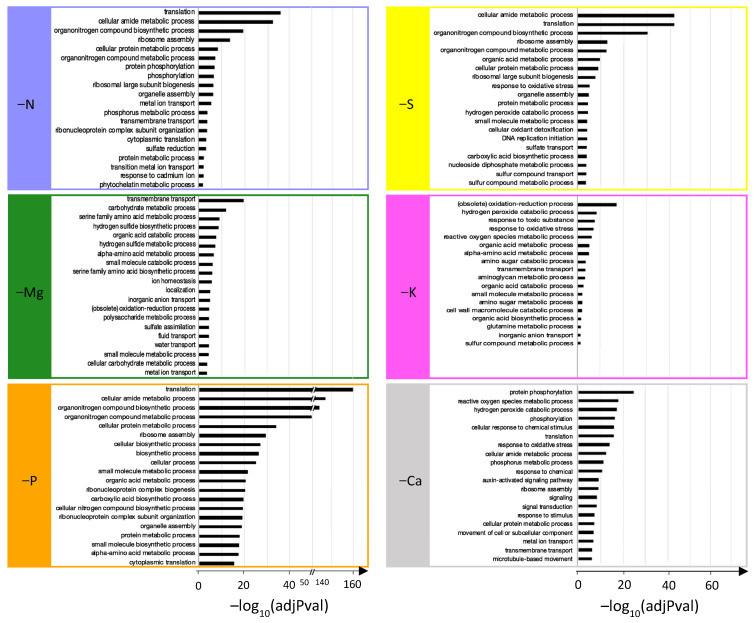
Top 20 significantly enriched “biological process” GO terms from DEGs of *Brassica napus* roots subjected to N, Mg, P, S, K, or Ca deprivations.

For each individual macronutrient deprivation, all enriched GO terms for “Biological Processes” were determined with g:Profiler and are provided in Supplemental Data SD4A. Redundant GO terms were removed with ReviGO and the top 20 enriched terms were kept and reported in Figure 2 for each macronutrient deprivation (except for −K, for which Revigo retrieved only 18 GO terms).

Interestingly, among the seven most enriched terms for the N, P, and S deprivations, six related to protein biosynthesis were common to all three deprivations (Figure 2: “translation”, “cellular amide metabolic process”, “organonitrogen compound biosynthetic process”, “ribosome assembly”, “cellular protein metabolic process”, and “organonitrogen compound metabolic process”). For N deprivation, other GO-enriched terms (Figure 2: “protein phosphorylation”, “phosphorylation”, “phosphorus metabolic process”, and “sulfate reduction”) highlighted interconnections of N metabolism with P and S metabolism. In addition to the seven most enriched GO terms, some found in P deprivation were also related to N metabolism (Figure 2: “cellular nitrogen compound biosynthetic process”, “protein metabolic process”, and “alpha amino acid metabolic process”). Surprisingly, processes related to P that were expected (Figure 2: “energetic metabolism” and “acid nucleic biosynthesis”) were less enriched (beyond the 20th rank; Supplemental Data SD4A). Similarly, GO terms related to S metabolism (“sulfate transport,” “sulfur compound transport”, and “sulfur compound metabolic process”) and ROS detoxification (“oxidative stress response”, “hydrogen peroxide”, “catabolic process”, and “cellular oxidant detoxification”) were enriched by S deprivation, but less so than those involved in N metabolism that were previously listed (Figure 2). Magnesium deprivation mainly led to the enrichment of GO terms related to “transport”, “photosynthesis”, “S metabolism”, and “amino acid metabolic processes” (Figure 2). Calcium deprivation enriched GO terms mainly associated with P metabolism (“protein phosphorylation”, “phosphorylation”, “phosphorus metabolic process”), signaling (“cellular response to chemical stimulus”, “response to chemical”, “auxin-activated signaling pathway”, “signaling”, “signal transduction”, “response to stimulus”), and ROS metabolic processes (“ROS metabolic process”, “hydrogen peroxide catabolic process”, “response to oxidative stress”). Finally, K deprivation massively enriched GO terms related to ROS (the top five most enriched) but also “organic acid” and “amino acid metabolic processes”, “transport”, and “sulfur compound metabolic process” (Figure 2).

**Figure 3:**
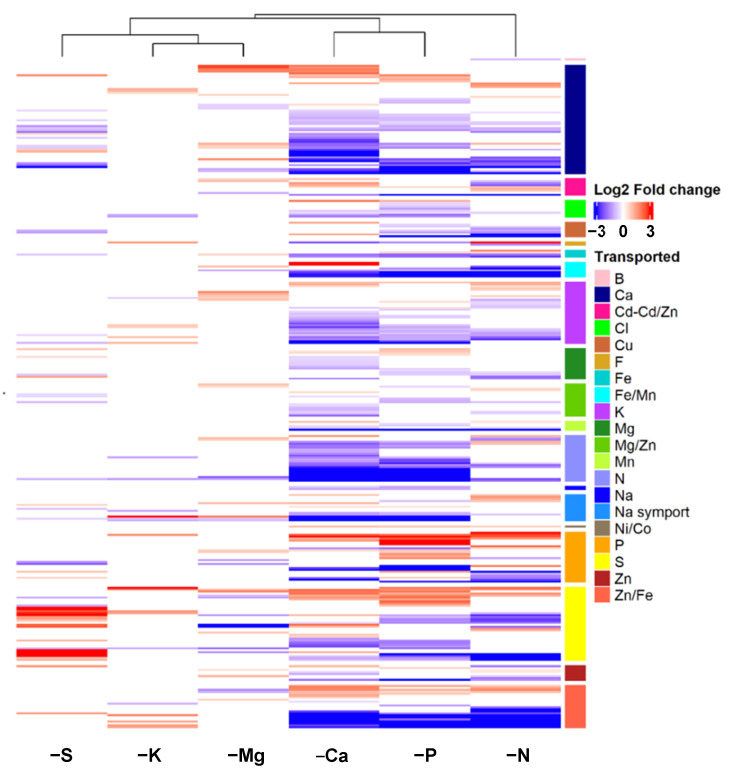
Log2 fold changes ratio of the 302 DEGs belonging to the “ion transport” group and clustered according to transported element. DEGs in roots of *Brassica napus* subjected to six individual macronutrient deprivations with significant (*p* < 0.05) upregulation (red) and downregulation (blue) compared to control plants.

It is interesting to note that the GO terms related to transport processes were enriched for all macronutrient deprivations, and were placed among the 20 most enriched GO terms for N, Mg, S, K, and Ca deprivations (Figure 2) and also in the 34th position (out of 65) for P deprivation (Supplemental Data SD4A). To further characterize the impacted transport processes, expression patterns of the 302 DEGs related to “ion transport” described in the Materials and Methods were analyzed (Figure 3). Irrespective of the macronutrient, each deprivation-modulated DEG related to the transport of a large panel of elements. However, the hierarchical clustering segregated the macronutrient deprivations into two groups: one that included N, Ca, and P deprivations, which strongly modulated genes encoding for all kinds of ion transporters, and a second that included S, K, and Mg deprivations, which modulated this panel of genes less broadly and less intensively. Finally, it is noteworthy that among the six macronutrients, only S and P deprivations led to a strong upregulation of genes encoding transporters involved in their own transport (Figure 3).

### 2.4. Identification and Functional Classification of DEGs Specific to a Single Macronutrient Deprivation

**Figure 4:**
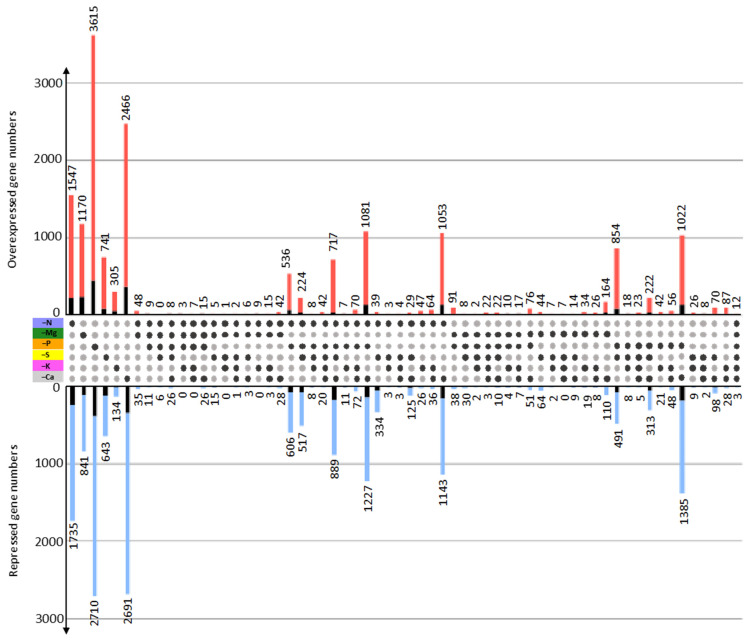
Number of upregulated (red) and downregulated (blue) DEGs specific to each macronutrient deprivation or common to n macronutrient deprivations (with *n* = 2 to 6) as indicated by the black dots. Black bars indicate the number of DEGs with “unknown” annotation.

By comparing the DEG profiles identified for each macronutrient deprivation, it was possible to determine DEGs with similar modulation among clusters of two to six macronutrient deprivations or specifically modulated by one macronutrient deprivation (i.e., modulated in only one macronutrient deprivation or modulated in an opposite direction by another macronutrient deprivation; Figure 4).

**Figure 5:**
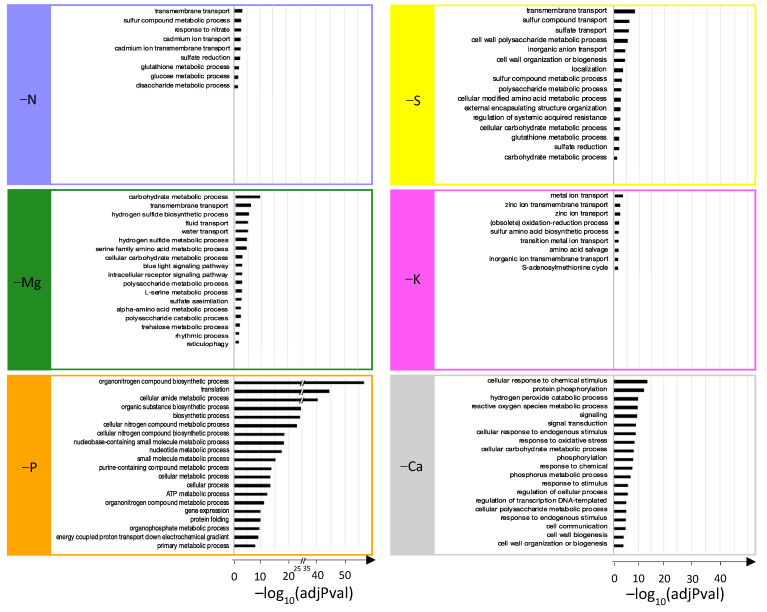
Top 20 significantly enriched “biological process” GO terms from specific DEGs of N, Mg, P, S, K, or Ca deprivations.

Looking at the genes that were common to *n* deprivations (2 < *n* < 6), the number of shared DEGs was dependent on the type of cluster being considered. For example, the N, P, and Ca macronutrient deprivation cluster shared 2308 (1081 up and 1227 down) DEGs, whereas the N, P, and K cluster only shared 18 (7 up and 11 down) DEGs, while only 15 DEGs (12 up and 3 down) were common to all six macronutrient deprivations. At a single deprivation level, 3282 (1547 up and 1735 down), 2011 (1170 up and 841 down), 6325 (3615 up and 2710 down), 1384 (741 up and 643 down), 439 (305 up and 134 down), and 5157 (2466 up and 2691 down) DEGs were specific to N, Mg, P, S, K, and Ca deprivation, respectively (Figure 4; Supplemental Data SD3). GO enrichment for “Biological Processes” was performed on sets of DEGs specific to each macronutrient deprivation (Supplemental Data SD4B). All enriched GO terms obtained for the N, Mg, S, and K deprivations (9, 18, 16, and 9, respectively) and a selection of the 20 most enriched GO terms for P and Ca deprivations are shown in Figure 5.

Despite an analysis focused on the DEGs specific to each macronutrient deprivation, very few biological processes unique to each element were identified, with the exception of the P and Ca deprivations, which affected energy metabolism (“ATP metabolic process”, “energy coupled proton transport down electrochemical gradient”) and signaling (“cellular response to chemical stimulus”, “signaling”, “signal transduction”, “cellular response to endogenous stimulus”, “response to chemical”, “response to stimulus” and “response to endogenous stimulus”), respectively. Indeed, the majority of enriched GO terms were related to ion transport, amino acids, carbohydrate, and, more importantly, sulfur metabolic processes, which were over-represented in five of the six macronutrient deprivations (Figure 5). Thus, enriched GO terms related to S metabolism were the following: “sulfur compounds metabolic process”, “sulfate reduction”, and “glutathione metabolic process” for N deprivation; “sulfur compound transport”, “sulfate transport”, “sulfur compounds metabolic process”, “glutathione metabolic process”, and “sulfate reduction” for S deprivation; “hydrogen sulfide biosynthetic process”, “hydrogen sulfide metabolic process”, and “sulfate assimilation” for Mg deprivation; “sulfur amino acid biosynthetic process” and “S-adenosylmethionine cycle” for K deprivation (Figure 5); and “sulfolipid metabolic process” and “sulfolipid biosynthetic process” for P deprivation (47th and 48th GO terms; Supplementary Data 4B). To illustrate this point more precisely, the relative expression levels and the functional annotations of DEGs specific to each macronutrient deprivation and related to S metabolism are presented in Table 2.

**Figure 6:**
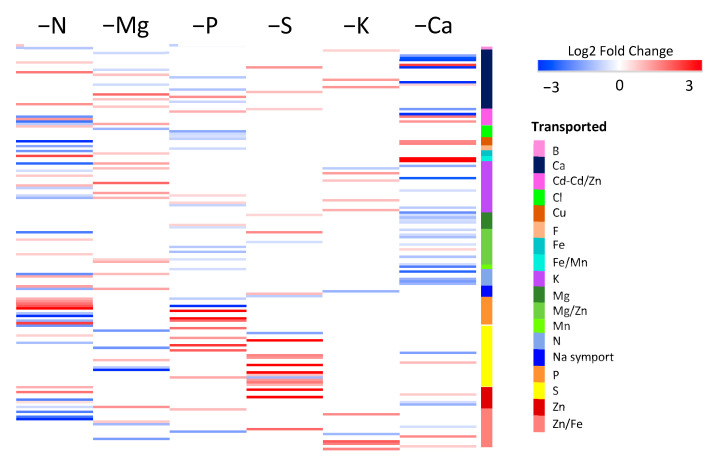
Log2 fold changes ratio of the specific DEGs belonging to the “ion transport” group. The red and blue scale indicates DEGs in roots of *Brassica napus* subjected to six individual macronutrient deprivations with upregulation and downregulation, respectively, compared to control plants.

In a general way, specific DEGs mainly related to S assimilation (PAPS reductase, ATP sulfurase, APS kinase, cysteine synthase, homocysteine S-methyltransferase, glutathione synthase, and glutathione S-transferase) were downregulated in N and Mg deprivation, while P and K deprivations upregulated DEGs encoding enzymes involved in sulfolipid biosynthesis (glycosyl transferase), cysteine biosynthesis (cysteine synthase), and methionine salvage (acireductone dioxygenase). Finally, S deprivation resulted in a strong upregulation of specific DEGs related to sulfate transport and S assimilation (APS kinase, PAPS reductase, methionine synthase, S adenosylmethionine synthetase), while several DEGs encoding glutathione S transferase were downregulated (Table 2). In the same way, GO enrichment performed with all specific DEGs highlighted terms related to ion transport (“transmembrane transport”, “cadmium ion transport”, “sulfur compound transport”, “sulfate transport”, “inorganic anion transport”, “metal ion transport”, “zinc ion transport”, “transition metal ion transport”) in four of the macronutrient deprivations (N, Mg, S, and K). Among DEGs specific to each macronutrient deprivation, the expression patterns of the 164 DEGs belonging to the set of 302 DEGs related to ion transport, as defined in the Material and Methods section, were analyzed (Figure 6 and Supplemental Data SD5).

Irrespective of the macronutrient deprivation, specific genes related to the transport of the deprived nutrient and/or other nutrient transports were modulated. Although only specific DEGs were considered, it was evident that the transport of Ca and Zn/Fe were affected by all macronutrient deprivations.

### 2.5. Metabolic Profiling of Individual Macronutrient Deprivations

**Figure 7:**
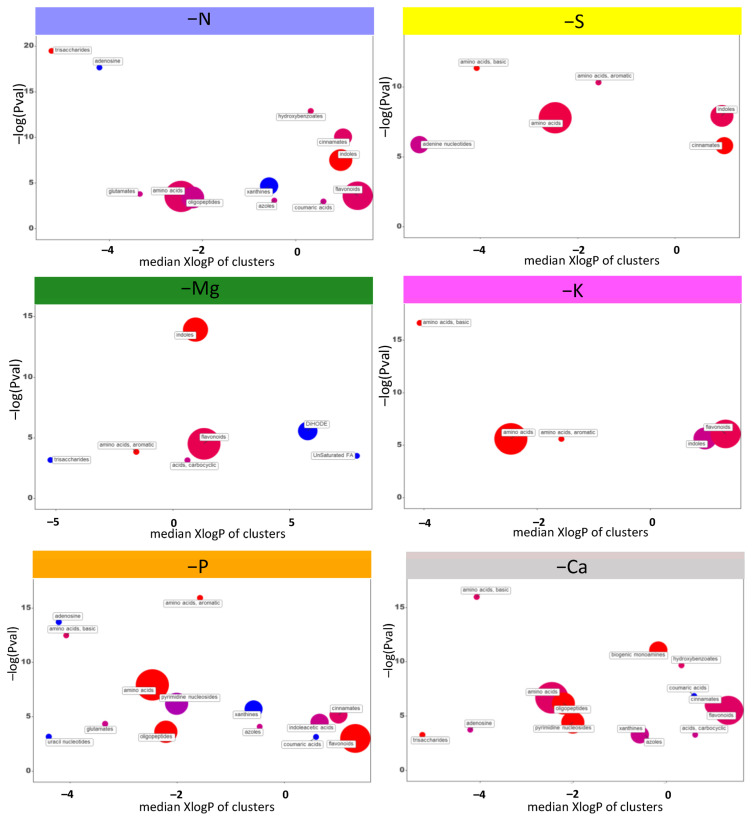
Chemical similarity clustering of the principal classes of metabolites performed by ChemRICH. Discs represent a significantly enriched family of modulated metabolites in *Brassica napus* roots subjected to six individual macronutrient deprivations. Enrichment −log(*p*-value) are displayed along the ordinate while the abscissa reflects the hydrophobicity (negative values) or hydrophilicity (positive values) of each group. Disc sizes varies with the total number of metabolites. Red, blue, and purple discs indicate groups with metabolites increased, decreased, and both increased and decreased, respectively.

Because transcriptomic analysis suggested that macronutrient deprivations broadly affected plant metabolism, an untargeted metabolomic approach was performed on roots of *Brassica napus*. For each macronutrient deficiency, a chemical similarity clustering of the principal classes of metabolites was performed by ChemRICH software (v4.0) using all the metabolites modulated compared to the control in order to highlight the main affected biological processes (Supplementary Data SD6; Figure 7).

**Figure 8:**
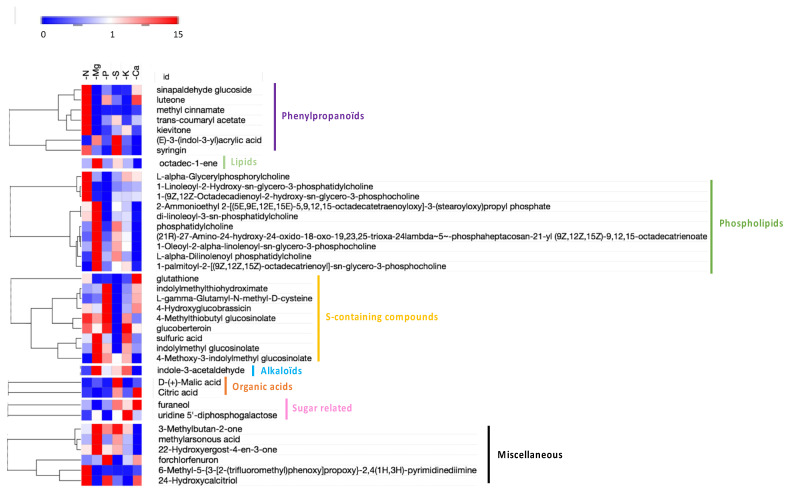
Hierarchical clustering of the 38 most discriminating metabolites (VIP > 4) significantly accumulated (red) and depleted (blue) relative to the control plants in *Brassica napus* roots subjected to six individual macronutrient deprivations. The colored dots indicate the biochemical classification of these metabolites.

Among the six macronutrient deprivations, K and S deprivations led to the lowest number of enriched metabolic classes with mainly “amino acids” and “indoles” for both, “flavonoids” for K deprivation, and “cinnamates” and “adenine nucleotides” for S deprivation. On the other hand, the N, P, and Ca deprivations were associated with the greatest enrichment of metabolic classes, among which it can be found again the “amino acids”, the “indoles”, and the “flavonoids”, but also several metabolic classes common to these three deprivations, such as “adenosine”, “xanthines”, “azoles”, “coumaric acids”, “oligopeptides”, and “cinnamates”. Interestingly, only Mg deprivation led to an enrichment of certain metabolic classes that were not found in any other deficiency. Thus, even though classes such as “amino acids” and “indoles” were once again highlighted, the “DIHODE” and “unsaturated fatty acids” classes were specifically enriched in response to Mg deprivation. Since there were few metabolic classes specifically enriched by one deprivation, an analysis at the metabolite scale was performed to try to discriminate the deprivations. Compared to the control, there were 451 metabolites overall that were significantly modulated (*p*-value < 0.001 and ratio >2) in at least one macronutrient deprivation, and these were further characterized (Supplementary Data SD7). From multivariate analysis, the 38 most discriminating metabolites (VIP > 4) for the six macronutrient deprivations were identified and assigned to major biochemical categories (Figure 8).

These discriminating compounds mainly belonged to “lipids”, “phospholipids”, “phenylpropanoids”, “S-containing compounds”, and, to a lesser extent, “organic acids”, “sugar related”, and “alkaloids”. Broadly, N deprivation mainly led to an accumulation of phenylpropanoid and a few phospholipid compounds, while Mg deprivation was principally associated with an accumulation of phospholipids and a decrease in phenylpropanoids. P deprivation led, among other things, to a decrease in phenylpropanoids and an accumulation of S-containing compounds, whereas S deprivation tended towards a decrease in these compounds. Under Ca deprivation, there was an accumulation of compounds belonging to different biochemical categories (one sugar related, one organic acid, one phenylpropanoid, and several S-containing compounds) and especially a strong decrease in phospholipids. Finally, K deprivation had the least impact on the metabolome, with only a decrease in some phenylpropanoids and an accumulation of one sugar-related and a few S-containing compounds.

## 3. Discussion

Maintaining the yield and quality of harvested products to support the food needs of the human population and reducing the use of inputs while managing the influence of climate change on nutrient availability requires a better understanding of plant responses to nutritional deficiencies and earlier diagnosis of their occurrence. This is especially true for crop species such as *Brassica napus*, which are highly demanding of fertilizers [42,43,44]. In this context, the objective of this study was to provide early insights into the major molecular changes in *Brassica napus* subjected to six individual macronutrient (N, Mg, P, S, K, and Ca) deprivations, with the aim of assessing them before they initiate large macroscopic changes (growth and photosynthesis). Consequently, molecular analysis were performed after ten days of deprivation when no decreases in root biomass or photosynthesis activity were observed, except for the P and N deprivations, respectively, where small changes were observed (Table 1; Supplementary Data SD8).

### 3.1. Macronutrient Deprivations Led to Profound Transcriptomic and Metabolomic Modifications in Roots

A large number of DEGS were found in roots under each of the macronutrient deprivations considered. A GO term enrichment analysis of these DEGs indicated that each macronutrient deprivation was associated with extensive changes, mostly related to the metabolism of the deprived nutrient, which was expected, but also related to other metabolic pathways and thus highlighting the interactions between elements. Compared to the other elements, K deprivation resulted in the smallest panel of DEGs (1470 DEGs, with 984 upregulated, and 486 downregulated), as previously observed in rice by [33]. This observation can be explained by the role of K as a monovalent cation mainly found in the vacuole that is especially involved in osmoregulation, rather than it participating in the direct biosynthesis of organic compounds [3,25]. Among the top five GO terms found in the enrichment analysis, four were related to oxidative responses. This is in agreement with several studies reporting strong links between ROS metabolism and K deficiency [45,46,47]. In contrast, P deprivation modulated the root transcriptome of *Brassica napus* the most, with changes in 17,387 DEGs (8839 upregulated and 8548 downregulated; Figure 1B) that belong to a large number of GO biological process terms mainly related to N metabolism (Figure 2), but also phosphorylation and energy metabolism (Supplementary Data SD4A). The effects of P deprivation on energy production [44,48,49] and its interactions with N metabolism are quite well documented [50]. Similarly, our study also highlighted these kinds of interactions in response to N deprivation. Indeed, N deprivation modulated 12,565 DEGs and in addition to the expected GO terms related to amino acid and proteins synthesis, there were enriched GO terms related to P metabolism such as “protein phosphorylation”, “phosphorylation”, and “phosphorus metabolic”. Similarly, the well-known N/S interaction [51,52] was also emphasized because S-related GO terms and N-related GO terms were observed under N and S deprivations, respectively. In addition, among the 3251 DEGs that responded to Mg deprivation, some of them were associated with C and N metabolisms [53] while others were related to S metabolism. Finally, with Ca being a well-known cellular messenger [22], it is not surprising that its deprivation generated a large panel of DEGs (15842), most of which were enriched GO terms related to signaling such as “protein phosphorylation”, thus highlighting a Ca/P interplay. These results are in agreement with previous studies performed in rice that indicated an association of Ca deprivation with the modulation of large panels of genes predominantly related to signaling [41].

Altogether, these GO enrichment analyses show that alongside metabolism and cellular processes directly related to the deprived nutrient, some other terms corresponding to more generic cellular processes were also systematically impacted. The best example is the GO term “ion transport”, which was enriched in response to all macronutrient deprivations (Figure 2 and Supplementary Data SD4B). Confirmation was given by the targeted analysis that focused on the 302 DEGs encoding ion transporters, which indicated that all macronutrient deprivations modulated the expression of genes encoding transporters for the deprived nutrients, and at the same time influenced genes belonging to other ion transporter categories (Figure 3). For example, P and S deprivations led to an upregulation of gene sets that encoded their own root transporters, as previously described in different plant species [11,54,55], but also a modulation of the expression of genes encoding other ion transporters (Figure 3). This wide-ranging modulation of the gene expression of numerous elemental transporters can be linked to recent studies performed in *Brassica napus* and *Triticum aestivum* where nutrient uptake and the element composition of plant tissues were broadly modified in response to a single macronutrient deprivation [9,11]. These generic responses observed in roots of plants subjected to six single macronutrient deprivations could be a consequence of the crucial roles of macronutrients in primary metabolism reported by many authors [3,12,13].

In addition, for each macronutrient deficiency, a metabolomic approach was implemented. The comparative analysis of metabolomic and transcriptomic modulations should have facilitated the deciphering of the biological processes involved in the response to macronutrient deprivation, but the overall connection between transcriptomic and metabolomic results remained elusive. This difficulty has been reported previously for nitrogen starvation in *Arabidopsis thaliana* by [56]. Nevertheless, in our study, chemical similarity clustering of major metabolite classes (Supplementary Data SD6 and Figure 7) revealed that the macronutrient deficiencies that resulted in the greatest number of enriched metabolite classes were also those with the greatest number of DEGS (N, P, and Ca deprivations; Figure 1B and Figure 7), and vice versa (S, Mg, and K deprivations; Figure 1B and Figure 7). Furthermore, all macronutrient deprivations led to an enrichment of the “amino acid” class, which is in agreement with a previous study also showing that P, K, Ca, or Mg macronutrient deficiencies all led to amino acid accumulation in bell pepper leaves and roots [57]. This accumulation of amino acids is consistent with GO enrichment analyses that show that all macronutrient deficiencies enriched protein synthesis and amino acid-related GO terms (“translation” and/or “ribosome” for N, S, P, and Ca deprivation and “metabolic/catabolic process of alpha amino acids” for Mg and K deprivation; Figure 2). It can be noticed that the enrichment analysis of metabolic similarities does not provide evidence for deprivation-specific metabolism. Indeed, with the exception of the metabolic classes “unsaturated fatty acids” and “DiHODE” that were specifically enriched in response to Mg deprivation, the other enriched metabolite classes (e.g., “amino acids”, “flavonoids”, “cinnamate”, “coumaric acid”, “indoles”, and “xanthine”) were always common to at least two deprivations.

### 3.2. Identification of Sets of DEGs and Metabolomic Profiles Specific to Each Macronutrient Deprivation

When considering the molecular responses to each macronutrient deprivation separately, it is not possible to identify a set of DEGs or metabolites specifically affected by each one. Only a few studies have compared the transcriptome response to several nutrient deprivations simultaneously, and even then, such work has been restricted to deficiencies in the three major macronutrients, N, P, and K [58,59]. To our knowledge, the only other work examining the effects of a large group of macronutrients is a study that was performed on 13 different nutrient availability anomalies in Arabidopsis [58]. However, even though this earlier study is relevant and provided new insights, the meta-analysis used had some limitations due to the dataset being derived from independent experiments that were performed with various genotypes, growing conditions, and treatment procedures. The strength of our study is the simultaneous deprivation of six individual macronutrients, which allowed us to carry out a comparative analysis of the molecular responses of each deprivation. Consequently, it was possible to identify DEGs common to several macronutrient deficiencies. As expected, those that shared the most DEGs (Figure 4, Supplementary Data SD3) were deprivations that involved macronutrients for which metabolic interactions were highlighted in the overall analysis discussed earlier (i.e., N, P, S, and/or Ca deprivations). In contrast, it was only when Mg and K deprivations were included that less common DEGs were found. More interestingly, this comparative analysis enabled the identification of a novel set of DEGs that were specifically upregulated and downregulated for each individual deprivation and for each of the six macronutrients (Figure 4, Supplementary Data SD3). In all cases, large sets of specific DEGs were identified (6325, 5157, 3282, 2011, 1384, and 439 for P, Ca, N, Mg, S, and K deprivation, respectively). Surprisingly, although these DEGs were specific to individual macronutrient deprivations, their GO enrichment analysis highlighted biological processes that were common to several macronutrient deprivations. This apparent discrepancy can be explained by the possibility that different specific DEGs may encode different enzymes involved in the same metabolic pathway or even encode different isoforms of the same enzyme.

To illustrate this, we focused on the S-related and transport biological process GO terms that were enriched in five and four out of the six macronutrient deprivations, respectively (Figure 4 and Figure 6). While a few specific DEGs encoded enzymes or transporters related to S that were found in only one macronutrient deprivation (e.g., glycosyl transferase or S transporter (SULTR) for P or S deprivation, respectively), numerous specific DEGs encoded different isoforms of the same enzyme found in several macronutrient deprivations, such as APS kinase in the N, Mg, and S deprivations (Table 2). This enrichment of the S metabolic pathways by specific DEGs for each macronutrient deprivation could be a consequence of the central role of S metabolism in the cell. Indeed, S metabolism provides cysteine for protein and glutathione synthesis, but also sulfated secondary metabolites involved in oxidative stress homeostasis in plants subjected to various stress conditions [60]. Considering specific DEGs encoding ion transporters, similar conclusions could be made because the transport of a given nutrient can be affected by several macronutrient deprivations through regulation of isoforms specific to each macronutrient deprivation. These two examples focusing on genes related to S and transport illustrate how several elements can interact in the transport and metabolism of other elements by modulating specific targets, and thus highlight the complexity of mineral interactions [3].

Similarly, the metabolic pathway enrichment analysis of all metabolic data focused on the 38 most discriminating metabolites (VIP > 4) but did not enable the identification of a specific metabolic pathway for any macronutrient deprivation (data not shown). Considering the biochemical classification and hierarchical clustering of the 38 most discriminating compounds, even though some trends seemed to emerge (e.g., loss of S-containing compounds and accumulation of phenylpropanoids under S and N deprivations, respectively), no clear evidence of a specific response to macronutrient deprivation could be found (Figure 8).

### 3.3. Towards Molecular Indicators to Discriminate Macronutrient Deficiencies

Diagnosis of a specific macronutrient deprivation can be challenging because the phenotypic and physiological responses are generally common [3], and therefore, there is little discriminating power. Over the past few decades, a handful of targeted transcriptomic approaches have been carried out [61,62,63,64], but they most often failed because the low number of gene candidates that were identified were modulated by deprivations in other macronutrients. To overcome this difficulty, one solution is to use a large-scale transcriptomic approach. This has been conducted by several authors, but most of the time, the limited number of macronutrient deprivations studied does not ensure that candidate genes are truly specific for a given macronutrient deprivation. The originality of the present study was to perform a comparative molecular analysis (transcriptomic and metabolomic) of six macronutrient deprivations (N, Mg, P, S, K, and Ca). For the first time, a large dataset of DEGs (amongst which 9844 were up-regulated, Figure 4) specific to each individual macronutrient deprivation is now available, from which a subset of specific DEGs could be extracted to detect each macronutrient deprivation. Concomitantly, this study also provides metabolomics analysis for the six macronutrient deprivations. Only a few metabolites seem to be specific to a macronutrient deprivation (methyl cinnamate, 6-methyl-5-{3-[2-(trifluoromethyl)phenoxy]propoxy}-2,4(1H,3H)-pyrimidinediimine, 1-linoleoyl-2-hydroxy-sn-glycero-3-phosphatidylcholine and 1-(9Z,12Z-octadecadienoyl-2-hydroxy-sn-glycero-3-phosphocholine for N deprivation; uridine 5′-diphosphogalactose for K deprivation; and d−(+)-malic acid for Ca deprivation; Figure 8). Nevertheless, taken together, the 38 most discriminating metabolites provide a specific metabolomic profile for the six individual macronutrient deprivations that could also be used as potential indicators of each macronutrient deprivation.

Results from our study constitute a relevant candidate database that foreshadows the development of a diagnostic tool for macronutrient deprivations. Nevertheless, numerous steps are still needed to reach this goal. This large data set of DEGs could be reduced, for example, by extracting those that are also expressed in other tissues such as leaves and considered generic if they are found in different cultivated species. Finally, it might be necessary to check the specificity of the molecular candidates when plants are faced with other biotic and abiotic stresses and/or multiple macronutrient deprivations. Ultimately, such a diagnostic tool could allow early and accurate detection of nutritional deficiencies in order to improve the management of field crop fertilization.

## 4. Material and Methods

### 4.1. Plant Material and Growth Conditions

Oilseed rape (*Brassica napus* cv. Trezzor) was grown in a greenhouse (20 °C day/15 °C night) in controlled hydroponic conditions. Seeds were germinated on perlite over demineralized water for five days in the dark and then under natural light until the first leaf appearance. At this stage, 10 seedlings were transferred into 10 L plastic containers (400 × 300 × 115 mm), each holding ten seedlings that were exposed to natural light supplemented by high-pressure sodium lamps (HPS 400 Watt, Hortilux Schreder, Monster, Netherlands), which, in combination, attained 350 μmol m^−2^ s^−1^ of photosynthetically active radiation for 16 h. As previously described by [9,10], the complete nutrient solution contained: 1 mM KNO_3_, 1.25 mM Ca(NO_3_)_2_, 0.2 mM KH_2_PO_4_, 0.4 mM MgSO_4_, 0.5 µM NaFe-EDTA, 50 µM NaFe-EDDHA, 10 μM H_3_BO_3_, 3 μM MnSO_4_, 3 μM ZnSO_4_, 0.7 μM CuSO_4_, 0.008 μM (NH_4_)_6_Mo_7_O_24_, 0.1 μM CoCl_2_, 0.15 μM NiCl_2_, 0.9 mM Si(OH)_4_, 0.5 mM CaCl_2_, 0.1 mM KCl, 0.01 µM Na_2_SeO_4_, 0.1 mM K_2_SO_4_, and 0.2 mM Na_2_SiO_3_ buffered to pH 6.8 with 0.36 mM CaCO_3_. The NO_3_^−^ concentration was monitored with nitrate test strips (Macherey-Nagel, Düren, Germany) in order to maintain optimal nutrition conditions. The nutrient solution was continuously aerated and renewed each time the NO_3_^−^ concentration reached thirty percent of its initial concentration.

After 24 days of growth with the complete nutrient solution, plants were separated into seven subsets: control plants, which were left in the complete nutrient solution, and six other groups that received a specific solution deprived of a single macronutrient (−N, −Mg, −P, −S, −K, or −Ca). These nutrient-deficient solutions adapted from the complete solution as previously described by [9] are detailed in Supplemental Table SD1. Throughout the deprivation treatments, photosynthetic activity was assessed every three days with a Li-6800 portable photosynthesis system (LI-COR, Lincoln, NE, United States) at 1000 µmol m^2^ s^−1^ of photon flux density (PFD) with chamber settings (i.e., temperature and relative humidity) matching environmental conditions.

For each treatment (control and macronutrient deprivations), five replicates of two individual plants were harvested after 10 days of macronutrient deprivation. This duration of privation was chosen according to [65] and [66] so that plants were harvested before a significant decrease in growth would occur in response to the macronutrient deprivation. After shoot and root fresh weight determination, root sample was split into two homogenous aliquots. One was frozen in liquid nitrogen and stored at −80 °C for transcriptomic and metabolomic analysis. The other was dried for 72 h at 70 °C for dry weight determination and elemental analysis performed with Isotopic-Ratio Mass Spectrometry (IRMS) for N and S and high-resolution Inductively Coupled Plasma Mass Spectrometry (ICP-MS) for all other elements, as previously described in Courbet et al. (2021).

### 4.2. RNA Extraction, Reverse Transcription and Q-PCR Analyses

Total RNAs were extracted from 200 mg of fresh root samples previously powdered using a mortar containing liquid nitrogen, according to the protocols of [67,68]. Briefly, 750 μL of hot phenol (80 °C, pH 4.3) and 750 μL of extraction buffer (0.1M TRIS, 0.1M LiCl, 0.01M EDTA, 1 % SDS (*w*/*v*), pH 8) were added and the mixture was vortexed for 40 s. Then, 750 μL of chloroform:isoamylalcohol (24/1: *v*/*v*) was added before centrifugation at 15,000× *g* for 5 min at 4 °C. The supernatant was recovered and 750 µL of a 4M LiCl solution (*w*/*v*) were added for nucleic acid precipitation overnight at 4 °C. The mixture was then centrifuged at 15,000× *g* for 20 min at 4 °C, the supernatant was removed, and 100 μL of sterile water was used to suspend the pellet. Extracted RNAs were purified by DNAse digestion using RNA Clean & Concentrator kits (Zymo Research, Irvine, CA, USA). Total RNA quantification was evaluated by spectrophotometry at 260 nm (BioPhotometer, Eppendorf, Le Pecq, France) before Reverse Transcription (RT). A 1 µg quantity of total RNAs was converted to cDNAs using an iScript cDNA synthesis kit (Bio-Rad, Marne-la-Coquette, France).

For qPCR, 4 µL of 100× diluted cDNAs were added to 11 µL of 1X SYBR Green Master Mix (Bio-Rad, Marne-la-Coquette, France) containing 0.5 µM of specific primers. Amplification reactions were performed with a real-time thermocycler (CFX96 Real Time System, Bio-Rad, Marne-la-Coquette, France) using the following three step program: an activation step at 95 °C for 3 min, 40 cycles of denaturation at 95 °C for 10 s, and finally, an extending step at 60 °C for 40 s. For q-PCR amplifications, the primers used are provided in Supplemental Table SD2A. For each pair of primers, threshold values and PCR efficiency (≈100%) were determined using a range of serial cDNA dilutions. The single peak in the melting curves and the sequencing of the amplicon (Eurofins, Nantes, France) validated the specificity of the amplification for each primer pair. Gene expression in the roots of macronutrient-deprived plants was calculated relative to the control with the ΔΔCt method using the following equation:

Relative expression = 2^−ΔΔCt^

With

ΔΔCt = ΔCt_sample_ − ΔCt_control_ (1)

and

ΔCt = Ct_target gene_ − Ct_housekeeping gene_ (2)

Using this method, root relative expression of the target gene in the control sample (without nutrient deprivation) was equal to 1 [69].

### 4.3. Transcriptomic Analysis by RNA-Sequencing (RNA-Seq)

The RNA-seq samples were obtained with Illumina NexSeq500 from POPS platform of Institute of Plant Science (IPS2) in Paris-Saclay (France). RNA-seq libraries were generated with the TruSeq Stranded mRNA protocol (Illumina^®^, California, CA, USA) with an average size of 260 bp and were sequenced in paired-end (PE) mode with a read length of 75 bases on the NextSeq500 with approximately 25 million PE reads per sample. To remove poor quality sequences, classical trimming (Qscore > 20, read length > 30) was performed and the STAR_2.5.2a mapper was used to align reads against the *Brassica napus* transcriptome (with local option and other default parameters). The abundance of each of the (annotation V5 from Genoscope accessed in February 2021: http://www.genoscope.cns.fr/brassicanapus/data/) was evaluated by unequivocal mapping of the PE reads to each gene. According to this method, 25% of reads were unmapped and 10% of reads with multi-hits were removed. Finally, 65% of reads may have combined with a gene without ambiguity.

Differential analysis of each gene followed the procedure described in [70]. Library size was normalized using the trimmed mean of M-values (TMM) method and count distribution was modeled with a negative binomial generalized linear model. Dispersion was estimated by the edgeR method (V1.12.0, [71]). Gene expression was compared between each macronutrient deprivation and control plants using the likelihood ratio test, and *p*-values were adjusted by the Benjamini–Hochberg procedure to control the False Discovery Rate (FDR, *p*-value < 0.05). We chose to consider a gene as differentially expressed (DEG) for an adjusted *p*-value ≤ 0.05, whatever the absolute value of the “Log2 fold change” (Supplementary Data SD3). Fragments Per Kilobase Million (FPKMs) were calculated for visual analysis only and were obtained by dividing normalized counts by gene length. PCA was performed with the FactoMineR 2.3 package under R (v 3.6.3) using log2-transformed normalized expression data.

RNA-seq expression data were validated by using eight DEGs with contrasting fold changes for RT-qPCR analysis. RT and qPCR were performed following the protocol described previously. Results of RT-qPCRs are presented in Supplemental Data SD2B.

### 4.4. RNA-Seq Bioinformatic Analysis

Gene ontology enrichment analysis for Differentially Expressed Genes (DEGs) was performed with gene ontology (GO) information slimmed to plants using g:Profiler (version e103_eg50_p15_eadf141) with the g:SCS multiple testing correction method (threshold of 0.05; [72]) and ReviGO (version February 01, 2021, with medium allowed similarity; [73]).

Among genes that are differentially expressed for at least one macronutrient deprivation, a set of *Brassica napus* DEGs was generated by filtering for the occurrence of the “transport” term in the annotation V5 from Genoscope. This list was supplemented with a set of DEGs associated with the GO term “ion transport” (GO:0006811) extracted from Amigo2 and unambiguously related to the transport of known elements (B, Ca, Cd, Cl, Co, Cu, F, Fe, K, Mg, Mn, N, Na, Ni, P, S, and Zn). This set of 302 DEGs encoding ion transporters is presented in Supplemental Data SD5.

### 4.5. Element Analysis by Mass Spectrometry and Uptake Calculation

As previously described by [9], dried samples were ground with 4 mm diameter inox beads using an oscillating grinder (Mixer Mill MM400, Retsch, Haan, Germany). Most macroelement (Mg, P, S, K, Ca) concentrations were quantified with 40 mg of dry powder previously subjected to mineralization, using high-resolution inductively coupled plasma mass spectrometry (HR-ICP-MS, Element 2^TM^, Thermo Fisher Scientific, Bremen, Germany) following the procedure described in [64].

Total N measurement was analyzed with 1.5 mg of fine powder placed in tin capsules before analysis with an isotope-ratio mass spectrometer (IRMS, Isoprime, GV Instruments, Manchester, U.K.) linked to a C/N/S analyzer (EA3000, Euro Vector, Milan, Italy).

Whole plant quantity (i.e., sum of all tissue quantities) was determined before the 10-day net uptake calculation as follow:

$$10\;day\;net\;uptake=\overset{n}{\underset{i\;1}{\sum }}Q_{Day\;10}-\overset{n}{\underset{i\;1}{\sum }}Q_{Day\;0}$$

where *n* = 5 tissues (roots, mature leaves, young leaves, mature petioles, young petioles) and Q = quantity of each element. This calculation was performed with consideration of all random combinations between the set of five replicates of Day 0 and Day 10. Thus, 10-day net uptake is indicated as the mean ± S.E for *n* = 25.

### 4.6. Untargeted Metabolic Profiling Using UPLC-MS/MS

Fifty milligrams of ground frozen root tissues were weighed and extracted with 1 mL of 70% MeOH (Optima LCMS grade, Fisher, U.K.), 29% H_2_O (Milli-Q, 18.2 MΩ·cm, Millipore, MA, USA) and 1% formic acid (LCMS grade, Fluka analytics, Germany). After extraction, samples were centrifuged, and the supernatant was collected for UPLC-MS/MS (ultra-performance liquid chromatography-tandem mass spectrometry) analysis. For the UPLC-MS/MS analysis, the separation and the detection were accomplished using an Acquity UPLC system (Waters, MA, USA) coupled to a Xevo G2-S QTof mass spectrometer (Waters) equipped with an LockSpray electrospray ionization (ESI) source. Sample separation was carried out by injecting 10 µL into an HSS T3 C18, 2.1 × 100 mm, 1.8 µm column (Waters) at a flow rate of 0.5 mL min^−1^, and the column oven was maintained at 40 °C. The mobile phases were composed of solvent A (Milli-Q water containing 0.1% formic acid) and solvent B (acetonitrile containing 0.1% formic acid). The separation was achieved by the following gradient: 0–1 min at 98% A, 1–7 min from 98% to 0% A, maintained at 0% A to 9 min, 9–10 min from 0% to 98% A, maintained at 98% until 12 min for column regeneration. The MS analysis was carried out in positive and negative ionization modes with the following parameters: source voltage 0.5 kV (pos) and 2.5 kV (neg); cone voltage 40 V; source temperature 130 °C; desolvation gas temperature 550 °C; desolvation gas flow 900 L/h. Mass spectra were acquired in MSE mode from 50 to 1200 *m*/*z* at 0.1 s scan^−1^. The ramp collision energy was set at 25 to 40 V. For each macronutrient deprivation, all metabolites significantly modulated compared to control are listed in Supplemental Data SD7. Compound IDs were added to each metabolites using ChemSpider. A chemical similarity enrichment analysis was performed using ChemRICH [74,75] which determines metabolite groups by hierarchical Tanimoto map. The *p*-value of each enriched group is given by the Kolmogorov-Smirnov test.

### 4.7. Statistical Analysis

Statistical analysis was based on five independent replicates each consisting of a pool of two individual plants, except for transcriptomic data, for which three independent replicates were used. Thus, plant biomass and photosynthesis are indicated as the mean ± S.E for *n* = 5, while net nutrient uptake was given as the mean ± S.E. for *n* = 25. Statistical analyses were performed using R software (v4.0.3) [76] and R Commander (v2.7-1). Significant differences between macronutrient deficient plants and control plants were determined using Student’s *t*-test. Heatmaps and clustering were generated with R Commander (v2.7-1) from the heatmap 2 package or the Morpheus-Broad Institute (https://software.broadinstitute.org). Regarding the metabolomics data, multivariate analysis was performed and allowed us to assign a variable importance in projection (VIP) score to each metabolite in order to determine the relative contribution of each metabolite to discriminate each macronutrient deficiency from the other.

## Figures and Tables

**Table 1:** Shoot and root dry weight, shoot/root ratio, photosynthesis, and net uptake of the deprived macronutrient by *Brassica napus* subjected or not (control) to six single macronutrient deprivation for ten days. Data are given as the mean ± SE. (*n* = 5 or *n* = 25 for uptake) and significant differences between control and macronutrient deprived plants are indicated (“*”: *p* < 0.01).

		Shoot Biomass (g plant^−1^)	Root Biomass (g plant^−1^)	Shoot/Root	Photosynthesis (µmol.CO_2_.m^−2^.s^−1^)	10-Days Uptake (mmol plant^−1^)	
						Macronutrient	
						Control	Deprived
	**Control**	5.56 ± 0.48	1.10 ± 0.04	5.05 ± 0.50	18.16 ± 1.47		
**Macronutrient deficiencies**	**−N**	4.47 ± 0.46	1.06 ± 0.09	4.20 ± 0.46	6.94 ± 1.29 *	N	
						9.44 ± 0.55	2.54 ± 0.44 *
	**−Mg**	6.06 ± 1.01	1.03 ± 0.15	5.86 ± 0.24	14.35 ± 2.74	Mg	
						0.66 ± 0.05	−0.019 ± 0.04 *
	**−P**	6.55 ± 0.46	1.44 ± 0.08 *	4.54 ± 0.35	12.74 ± 1.36 *	P	
						0.72 ± 0.05	0.02 ± 0.01 *
	**−S**	6.35 ± 0.88	1.11 ± 0.09	5.74± 0.0.31	19.39 ± 1.35	S	
						1.62 ± 0.10	0.26 ± 0.06 *
	**−K**	5.91 ± 0.55	1.12 ± 0.16	5.30 ± 0.54	21.50 ± 1.41	K	
						6.94 ± 0.36	0.54 ± 0.21 *
	**−Ca**	5.82 ± 0.33	1.10 ± 0.12	5.28± 0.44	19.86 ± 1.94	Ca	
						3.12 ± 0.22	0.05 ± 0.01 *

**Table 2:** Identification (ID) and annotation of significantly DEGs (*p* < 0.05)*,* specific to a macronutrient deprivation and related to S metabolic pathways (KEGG pathway ID) and sulfate transport. Up- (red) and down-regulation (blue) compared to control plants.

	Genoscope Gene ID	Gene ID	Protein ID	KEGG Pathway	Annotation	log2FC	Padj
−**N**	*BnaC04g19270D*	*GSBRNA2T00124039001*	CDY07244	bna00920	PAPS reductase	−1.00	2.8 × 10^−2^
	*BnaA03g59800D*	*GSBRNA2T00028621001*	CDY24876	bna00920		−1.88	1.9 × 10^−5^
	*BnaA01g11840D*	*GSBRNA2T00131663001*	CDX79117	bna00920		−1.97	1.1 × 10^−8^
	*BnaA09g20370D*	*GSBRNA2T00094497001*	CDY16659	bna00920		−2.52	1.8 × 10^−25^
	*BnaA03g45080D*	*GSBRNA2T00106793001*	CDX98723	bna00920		−2.13	1.4 × 10^−15^
	*BnaCnng62800D*	*GSBRNA2T00086154001*	CDY69279	bna00920	ATP sulfurylase	−1.47	3.8 × 10^−6^
	*BnaA03g36860D*	*GSBRNA2T00154839001*	CDX92964	bna00920		−1.59	2.2 × 10^−9^
	*BnaC01g22950D*	*GSBRNA2T00090739001*	CDY16260	bna00920		−1.62	1.6 × 10^−2^
	*BnaC07g51290D*	*GSBRNA2T00039753001*	CDY63923	bna00920	APS kinase	−1.16	3.5 × 10^−2^
	*BnaA03g54400D*	*GSBRNA2T00050135001*	CDY31612	bna00920		−1.22	1.3 × 10^−2^
	*BnaCnng62620D*	*GSBRNA2T00084257001*	CDY69239	bna00920/bna00270/bna01230	Cysteine synthase	0.71	3.7 × 10^−2^
	*BnaC07g27640D*	*GSBRNA2T00133834001*	CDX80680	bna00920/bna00270/bna01230		−2.48	2.6 × 10^−3^
	*BnaA09g55670D*	*GSBRNA2T00019599001*	CDY56449	bna00270	Homocysteine S-methyltransferase	−0.77	8.8 × 10^−4^
	*BnaA06g33260D*	*GSBRNA2T00078007001*	CDY13597	bna00270		−0.91	3.8 × 10^−2^
	*BnaC09g03120D*	*GSBRNA2T00109460001*	CDX99989	bna00480	Glutathione synthase	−0.57	3.4 × 10^−3^
	*BnaA09g14510D*	*GSBRNA2T00081552001*	CDY45141	bna00480	Glutathione S-transferase	1.99	7.5 × 10^−7^
	*BnaA09g14520D*	*GSBRNA2T00081550001*	CDY45140	bna00480		1.37	1.3 × 10^−3^
	*BnaA09g29540D*	*GSBRNA2T00090678001*	CDY16220	bna00480		−1.17	3.1 × 10^−2^
	*BnaC06g25030D*	*GSBRNA2T00022180001*	CDY23534	bna00480		−1.62	2.9 × 10^−2^
	*BnaA07g09120D*	*GSBRNA2T00069459001*	CDY40288	bna00480		−1.67	2.8 × 10^−2^
−**Mg**	*BnaC03g48570D*	*GSBRNA2T00062167001*	CDY36498	bna00920	APS kinase	2.08	2.2 × 10^−13^
	*BnaA06g25000D*	*GSBRNA2T00000648001*	CDY08724	bna00920		1.57	1.3 × 10^−9^
	*BnaA03g38670D*	*GSBRNA2T00155065001*	CDX93145	bna00920		−1.20	5.2 × 10^−3^
	*BnaCnng47170D*	*GSBRNA2T00046596001*	CDY65440	bna00920		−1.36	1.6 × 10^−3^
	*BnaC01g00790D*	*GSBRNA2T00131229001*	CDX69374	bna00920		−1.43	3.3 × 10^−2^
	*BnaA05g37220D*	*GSBRNA2T00097688001*	CDY50861	bna00920		−1.53	6.3 × 10^−5^
	*BnaA01g34620D*	*GSBRNA2T00039722001*	CDY63896	bna00920		−1.78	1.6 × 10^−2^
−**P**	*BnaA10g27550D*	*GSBRNA2T00069492001*	CDY40313	bna01100	Glycosyl transferase	6.39	5.4 × 10^−67^
	*BnaA10g27560D*	*GSBRNA2T00069491001*	CDY40312	bna01100	Glycosyl transferase	5.68	7.8 × 10^−29^
	*BnaCnng03870D*	*GSBRNA2T00032420001*	CDY10486	bna01100	Glycosyl transferase	5.01	1.7 × 10^−76^
	*BnaA03g00460D*	*GSBRNA2T00049228001*	CDY11210	bna01100	Glycosyl transferase	2.31	2.2 × 10^−17^
−**S**	*BnaC07g18000D*	*GSBRNA2T00158266001*	CDX94791		SULTR1.1	7.70	1.3 × 10^−14^
	*BnaA02g10510D*	*GSBRNA2T00036843001*	CDY27031		SULTR1.1	6.15	1.2 × 10^−111^
	*BnaC02g14670D*	*GSBRNA2T00102253001*	CDX96184		SULTR1.1	5.15	2.5 × 10^−68^
	*BnaA10g22050D*	*GSBRNA2T00135917001*	CDX69856		SULTR2.1	3.90	5.4 × 10^−12^
	*BnaC09g46440D*	*GSBRNA2T00103928001*	CDX96981		SULTR2.1	3.77	4.0 × 10^−4^
	*BnaC06g38470D*	*GSBRNA2T00147936001*	CDX88403		SULTR2.2	1.62	6.6 × 10^−11^
	*BnaA07g33850D*	*GSBRNA2T00146476001*	CDX87488		SULTR2.2	1.46	1.3 × 10^−6^
	*BnaA07g33860D*	*GSBRNA2T00146474001*	CDX87487		SULTR1.2	1.27	1.4 × 10^−2^
	*BnaC05g18450D*	*GSBRNA2T00119410001*	CDY04744		Sulphate anion transporter	1.24	3.4 × 10^−2^
	*BnaC06g38480D*	*GSBRNA2T00147937001*	CDX88404		SULTR1.2	0.89	4.3 × 10^−2^
	*BnaC03g05940D*	*GSBRNA2T00140339001*	CDX70510		SULTR4.1	0.73	1.9 × 10^−2^
	*BnaC03g39450D*	*GSBRNA2T00123837001*	CDX75862		Sulphate anion transporter	−1.06	4.2 × 10^−2^
	*BnaC09g08710D*	*GSBRNA2T00135048001*	CDX81460	bna00920	APS kinase	0.99	6.0 × 10^−5^
	*BnaA09g08410D*	*GSBRNA2T00063703001*	CDY37220	bna00920		0.91	2.0 × 10^−4^
	*BnaC01g13420D*	*GSBRNA2T00138265001*	CDX82892	bna00920	PAPS reductase	1.31	5.9 × 10^−3^
	*BnaA03g45080D*	*GSBRNA2T00106793001*	CDX98723	bna00920		1.07	5.3 × 10^−4^
	*BnaA09g20370D*	*GSBRNA2T00094497001*	CDY16659	bna00920		0.84	2.1 × 10^−3^
	*BnaC07g37060D*	*GSBRNA2T00156974001*	CDX94072	bna00920		0.62	2.8 × 10^−2^
	*BnaC09g39920D*	*GSBRNA2T00032694001*	CDY25477	bna01230/bna00270	Methionine synthase	0.57	3.0 × 10^−2^
	*BnaA03g34510D*	*GSBRNA2T00137491001*	CDX82370	bna01230/bna00270	S-adenosylmethionine synthetase	0.87	2.2 × 10^−2^
	*BnaC06g41890D*	*GSBRNA2T00023030001*	CDY58045	bna00480	Glutathione S-transferase	1.31	4.2 × 10^−3^
	*BnaA06g20200D*	*GSBRNA2T00061438001*	CDY12324	bna00480		0.71	1.0 × 10^−3^
	*BnaC05g13360D*	*GSBRNA2T00091169001*	CDY48476	bna00480		−0.68	1.1 × 10^−2^
	*BnaA06g11500D*	*GSBRNA2T00034941001*	CDY26267	bna00480		−1.04	1.2 × 10^−3^
	*BnaA04g28590D*	*GSBRNA2T00003272001*	CDY51365	bna00480		−1.29	4.0 × 10^−2^
	*BnaA06g11510D*	*GSBRNA2T00034940001*	CDY26266	bna00480		−1.37	2.8 × 10^−2^
	*BnaC05g13350D*	*GSBRNA2T00091168001*	CDY48475	bna00480		−1.89	6.0 × 10^−5^
−**K**	*BnaAnng28500D*	*GSBRNA2T00080405001*	CDY68838	bna00270/bna01230	Cysteine synthase	0.98	1.4 × 10^−3^
	*BnaA07g04420D*	*GSBRNA2T00054274001*	CDY33397	bna00270	Initiation factor 2B-related	0.75	1.2 × 10^−3^
	*BnaA01g19100D*	*GSBRNA2T00043605001*	CDY29756	bna00270	Acireductone dioxygenase	0.73	1.6 × 10^−3^
	*BnaCnng67980D*	*GSBRNA2T00098221001*	CDY70356	bna00270	Initiation factor 2B-related	0.47	4.4 × 10^−2^

## Data Availability

RNAseq data were submitted to the Gene Expression Omnibus (GEO) international repository: http://www.ncbi.nlm.nih.gov/geo; GEO accession: GSE180378.

## Supplementary Materials

The following are available online at https://www.mdpi.com/article/10.3390/ijms222111679/s1.

## References

[1] Amtmann A., Armengaud P. (2009). Effects of N, P, K and S on metabolism: New knowledge gained from multi-level analysis. Curr. Opin. Plant Biol..

[2] O’Rourke J.A., McCabe C.E., Graham M.A. (2020). Dynamic gene expression changes in response to micronutrient, macronutrient, and multiple stress exposures in soybean. Funct. Integr. Genom..

[3] De Bang T.C., Husted S., Laursen K.H., Persson D.P., Schjoerring J.K. (2021). The molecular–physiological functions of mineral macronutrients and their consequences for deficiency symptoms in plants. New Phytol..

[4] Silva E.D.B., Santos A.A., De Mattos A.M., Neto A.M.B., Da Cruz M.D.C.M., Moreira R.A., Júnior V.C.D.A., Gonçalves E.D., De Oliveira L.F. (2017). Visual symptoms of nutrient deficiencies in *Physalis peruviana* L. Biosci. J..

[5] Koné B., Emile Y., Amadji G.L., N’Ganzou K.R. (2014). Root and Grain Yield as Affected by Soil Nutrient Deficiency and Fertilizer in Rainfed Rice Cultivation. Advanced fertilizer technology Syntheis.

[6] Tränkner M., Jákli B., Tavakol E., Geilfus C.-M., Cakmak I., Dittert K., Senbayram M. (2016). Magnesium deficiency decreases biomass water-use efficiency and increases leaf water-use efficiency and oxidative stress in barley plants. Plant Soil.

[7] Baxter I.R., Vitek O., Lahner B., Muthukumar B., Borghi M., Morrissey J., Guerinot M.L., Salt D.E. (2008). The leaf ionome as a multivariable system to detect a plant’s physiological status. Proc. Natl. Acad. Sci. USA.

[8] Baxter I. (2009). Ionomics: Studying the social network of mineral nutrients. Curr. Opin. Plant Biol..

[9] Courbet G., D’Oria A., Lornac A., Diquélou S., Pluchon S., Arkoun M., Koprivova A., Kopriva S., Etienne P., Ourry A. (2021). Specificity and Plasticity of the Functional Ionome of *Brassica napus* and *Triticum aestivum* Subjected to Macronutrient Deprivation. Front. Plant Sci..

[10] D’Oria A., Courbet G., Lornac A., Pluchon S., Arkoun M., Maillard A., Etienne P., Diquélou S., Ourry A. (2021). Specificity and Plasticity of the Functional Ionome of *Brassica napus* and *Triticum aestivum* Exposed to Micronutrient or Beneficial Nutrient Deprivation and Predictive Sensitivity of the Ionomic Signatures. Front. Plant Sci..

[11] Maillard A., Etienne P., Diquélou S., Trouverie J., Billard V., Yvin J.-C., Ourry A. (2016). Nutrient deficiencies modify the ionomic composition of plant tissues: A focus on cross-talk between molybdenum and other nutrients in *Brassica napus*. J. Exp. Bot..

[12] Maathuis F.J. (2009). Physiological functions of mineral macronutrients. Curr. Opin. Plant Biol..

[13] Hawkesford M., Horst W., Kichey T., Lambers H., Schjoerring J., Møller I.S., White P., Marschner P. (2012). Chapter 6-Functions of Macronu-trients. Marschner’s Mineral Nutrition of Higher Plants.

[14] Desclos M., Etienne P., Coquet L., Jouenne T., Bonnefoy J., Segura R., Reze S., Ourry A., Avice J.-C. (2009). A combined ^15^N tracing/proteomics study in *Brassica napus* reveals the chronology of proteomics events associated with N remobilisation during leaf senescence induced by nitrate limitation or starvation. Proteomics.

[15] Onodera J., Ohsumi Y. (2005). Autophagy Is Required for Maintenance of Amino Acid Levels and Protein Synthesis under Nitrogen Starvation. J. Biol. Chem..

[16] Hawkesford M.J. (2000). Plant responses to sulphur deficiency and the genetic manipulation of sulphate transporters to improve S-utilization efficiency. J. Exp. Bot..

[17] Davidian J.-C., Kopriva S. (2010). Regulation of Sulfate Uptake and Assimilation—the Same or Not the Same?. Mol. Plant.

[18] Kopriva S., Malagoli M., Takahashi H. (2019). Sulfur nutrition: Impacts on plant development, metabolism, and stress responses. J. Exp. Bot..

[19] Willows R.D. (2003). Biosynthesis of chlorophylls from protoporphyrin IX. Nat. Prod. Rep..

[20] Lambers H., Finnegan P., Jost R., Plaxton W., Shane M.W., Stitt M. (2015). Phosphorus nutrition in Proteaceae and beyond. Nat. Plants.

[21] Hocking B., Tyerman S.D., Burton R.A., Gilliham M. (2016). Fruit Calcium: Transport and Physiology. Front. Plant Sci..

[22] Aldon D., Mbengue M., Mazars C., Galaud J.-P. (2018). Calcium Signalling in Plant Biotic Interactions. Int. J. Mol. Sci..

[23] Thor K. (2019). Calcium—Nutrient and Messenger. Front. Plant. Sci..

[24] Leigh R.A., Wyn Jones R.G. (1984). A hypothesis relating critical potassium concentrations for growth to the distribution and functions of this ion in the plant cell. New Phytol..

[25] Cushman J.C. (2001). Osmoregulation in Plants: Implications for Agriculture. Am. Zoöl..

[26] Hafsi C., Debez A., Abdelly C. (2014). Potassium deficiency in plants: Effects and signaling cascades. Acta Physiol. Plant..

[27] Yang W., Yoon J., Choi H., Fan Y., Chen R., An G. (2015). Transcriptome analysis of nitrogen-starvation-responsive genes in rice. BMC Plant. Biol..

[28] Curci P.L., Cigliano R.A., Zuluaga D.L., Janni M., Sanseverino W., Sonnante G. (2017). Transcriptomic response of durum wheat to nitrogen starvation. Sci. Rep..

[29] Nikiforova V., Freitag J., Kempa S., Bielecka M., Hesse H., Hoefgen R. (2003). Transcriptome analysis of sulfur depletion inArabidopsis thaliana: Interlacing of biosynthetic pathways provides response specificity. Plant. J..

[30] Bielecka M., Watanabe M., Morcuende R., Scheible W.-R., Hawkesford M., Hesse H., Hoefgen R. (2015). Transcriptome and metabolome analysis of plant sulfate starvation and resupply provides novel information on transcriptional regulation of metabolism associated with sulfur, nitrogen and phosphorus nutritional responses in Arabidopsis. Front. Plant. Sci..

[31] Yu Z., Juhasz A., Islam S., Diepeveen D., Zhang J., Wang P., Ma W. (2018). Impact of mid-season sulphur deficiency on wheat nitrogen metabolism and biosynthesis of grain protein. Sci. Rep..

[32] Canales J., Uribe F., Henríquez-Valencia C., Lovazzano C., Medina J., Vidal E.A. (2020). Transcriptomic analysis at organ and time scale reveals gene regulatory networks controlling the sulfate starvation response of *Solanum lycopersicum*. BMC Plant. Biol..

[33] Ma T.-L., Wu W.-H., Wang Y. (2012). Transcriptome analysis of rice root responses to potassium deficiency. BMC Plant. Biol..

[34] Armengaud P., Breitling R., Amtmann A. (2004). The Potassium-Dependent Transcriptome of Arabidopsis Reveals a Prominent Role of Jasmonic Acid in Nutrient Signaling. Plant. Physiol..

[35] Wang Y., Li B., Du M.W., Eneji A.E., Wang B.M., Duan L.S., Li Z.H., Tian X.L. (2012). Mechanism of phytohormone involvement in feedback regulation of cotton leaf senescence induced by potassium deficiency. J. Exp. Bot..

[36] Zeng J., He X., Wu D., Zhu B., Cai S., Nadira U.A., Jabeen Z., Zhang G. (2014). Comparative Transcriptome Profiling of Two Tibetan Wild Barley Genotypes in Responses to Low Potassium. PLoS ONE.

[37] He Y., Li R., Lin F., Xiong Y., Wang L., Wang B., Guo J., Hu C. (2019). Transcriptome Changes Induced by Different Potassium Levels in Banana Roots. Plants.

[38] Sega P., Kruszka K., Bielewicz D., Karlowski W., Nuc P., Szweykowska-Kulinska Z., Pacak A. (2021). Pi-starvation induced transcriptional changes in barley revealed by a comprehensive RNA-Seq and degradome analyses. BMC Genom..

[39] Mo X., Zhang M., Liang C., Cai L., Tian J. (2019). Integration of metabolome and transcriptome analyses highlights soybean roots responding to phosphorus deficiency by modulating phosphorylated metabolite processes. Plant. Physiol. Biochem..

[40] Yang L.-T., Zhou Y.-F., Wang Y.-Y., Wu Y.-M., Ye X., Guo J.-X., Chen L.-S. (2019). Magnesium Deficiency Induced Global Transcriptome Change in *Citrus sinensis* Leaves Revealed by RNA-Seq. Int. J. Mol. Sci..

[41] Shankar A., Srivastava A.K., Yadav A.K., Sharma M., Pandey A., Raut V.V., Das M.K., Suprasanna P., Pandey G.K. (2014). Whole genome transcriptome analysis of rice seedling reveals alterations in Ca^2+^ ion signaling and homeostasis in response to Ca^2+^ deficiency. Cell Calcium.

[42] Akmouche Y., Cheneby J., Lamboeuf M., Elie N., Laperche A., Bertheloot J., D’Hooghe P., Trouverie J., Avice J.-C., Etienne P. (2019). Do nitrogen- and sulphur-remobilization-related parameters measured at the onset of the reproductive stage provide early indicators to adjust N and S fertilization in oilseed rape (*Brassica napus* L.) grown under N- and/or S-limiting supplies?. Planta.

[43] Poisson E., Trouverie J., Brunel-Muguet S., Akmouche Y., Pontet C., Pinochet X., Avice J.-C. (2019). Seed Yield Components and Seed Quality of Oilseed Rape Are Impacted by Sulfur Fertilization and Its Interactions With Nitrogen Fertilization. Front. Plant. Sci..

[44] Tian J., Wang C., Zhang Q., He X., Whelan J., Shou H. (2012). Overexpression ofOsPAP10a, A Root-Associated Acid Phosphatase, Increased Extracellular Organic Phosphorus Utilization in Rice. J. Integr. Plant. Biol..

[45] Kim M.J., Ciani S., Schachtman D.P. (2010). A Peroxidase Contributes to ROS Production during Arabidopsis Root Response to Potassium Deficiency. Mol. Plant..

[46] DU Q., Zhao X.-H., Xia L., Jiang C.-J., Wang X.-G., Han Y., Wang J., Yu H.-Q. (2019). Effects of potassium deficiency on photosynthesis, chloroplast ultrastructure, ROS, and antioxidant activities in maize (*Zea mays* L.). J. Integr. Agric..

[47] Behera S., Long Y., Schmitz-Thom I., Wang X., Zhang C., Li H., Steinhorst L., Manishankar P., Ren X., Offenborn J.N. (2017). Two spatially and temporally distinct Ca^2+^ signals convey *Arabidopsis thaliana* responses to K + deficiency. New Phytol..

[48] Hammond J.P., Broadley M., White P. (2004). Genetic Responses to Phosphorus Deficiency. Ann. Bot..

[49] Malhotra H., Sharma S., Pandey R., Hasanuzzaman M., Fujita M., Oku H., Nahar K., Hawrylak-Nowak B. (2018). Phosphorus nutrition: Plant growth in response to deficiency and excess. Plant Nutrients and Abiotic Stress Tolerance.

[50] Krouk G., Kiba T. (2020). Nitrogen and Phosphorus interactions in plants: From agronomic to physiological and molecular insights. Curr. Opin. Plant. Biol..

[51] Jamal A., Moon Y.-S., Zainul Abdin M. (2010). Sulphur-a General Overview and Interaction with Nitrogen. Aust. J. Crop. Sci..

[52] Kopriva S. (2004). Control of sulphate assimilation and glutathione synthesis: Interaction with N and C metabolism. J. Exp. Bot..

[53] Guo W., Nazim H., Liang Z., Yang D. (2016). Magnesium deficiency in plants: An urgent problem. Crop. J..

[54] Aarabi F., Naake T., Fernie A.R., Hoefgen R. (2020). Coordinating Sulfur Pools under Sulfate Deprivation. Trends Plant. Sci..

[55] Aziz T., Sabir M., Farooq M., Maqsood M.A., Ahmad H.R., Warraich E.A. (2014). Phosphorus Deficiency in Plants: Responses, Adaptive Mechanisms, and Signaling. Plant Signaling: Understanding the Molecular Crosstalk.

[56] Krapp A., Berthomé R., Orsel M., Mercey-Boutet S., Yu A., Castaings L., Elftieh S., Major H., Renou J.-P., Daniel-Vedele F. (2011). Arabidopsis Roots and Shoots Show Distinct Temporal Adaptation Patterns toward Nitrogen Starvation. Plant. Physiol..

[57] Kim Y.X., Kim T.J., Lee Y., Lee S., Lee D., Oh T.-K., Sung J. (2018). Metabolite profiling and mineral nutrient analysis from the leaves and roots of bell pepper (*Capsicum annuum* L. var. *angulosum*) grown under macronutrient mineral deficiency. Appl. Biol. Chem..

[58] Takehisa H., Sato Y., Antonio B., Nagamura Y. (2015). Coexpression Network Analysis of Macronutrient Deficiency Response Genes in Rice. Rice.

[59] Zhu Z., Li D., Wang P., Li J., Lu X. (2020). Transcriptome and ionome analysis of nitrogen, phosphorus and potassium interactions in sorghum seedlings. Theor. Exp. Plant. Physiol..

[60] Brumbarova T., Ivanov R. (2019). The Nutrient Response Transcriptional Regulome of Arabidopsis. iScience.

[61] Chan K.X., Phua S.Y., Van Breusegem F. (2019). Secondary sulfur metabolism in cellular signalling and oxidative stress responses. J. Exp. Bot..

[62] Shigaki T., Hirschi K. (2000). Characterization of CAX-like genes in plants: Implications for functional diversity. Gene.

[63] Hermans C., Vuylsteke M., Coppens F., Cristescu S.M., Harren F.J.M., Inzé D., Verbruggen N. (2010). Systems analysis of the responses to long-term magnesium deficiency and restoration in *Arabidopsis thaliana*. New Phytol..

[64] Kamiya T., Yamagami M., Hirai M., Fujiwara T. (2011). Establishment of an in planta magnesium monitoring system using CAX3 promoter-luciferase in Arabidopsis. J. Exp. Bot..

[65] Verbruggen N., Hermans C. (2013). Physiological and molecular responses to magnesium nutritional imbalance in plants. Plant. Soil.

[66] Maillard A., Diquélou S., Billard V., Laîné P., Garnica M., Prudent M., Garcia-Mina J.-M., Yvin J.-C., Ourry A. (2015). Leaf mineral nutrient remobilization during leaf senescence and modulation by nutrient deficiency. Front. Plant. Sci..

[67] Sorin E., Etienne P., Maillard A., Zamarreño A.-M., Garcia-Mina J.-M., Arkoun M., Jamois F., Cruz F., Yvin J.-C., Ourry A. (2015). Effect of sulphur deprivation on osmotic potential components and nitrogen metabolism in oilseed rape leaves: Identification of a new early indicator. J. Exp. Bot..

[68] Haddad C., Arkoun M., Jamois F., Schwarzenberg A., Yvin J.-C., Etienne P., Laîné P. (2018). Silicon Promotes Growth of *Brassica napus* L. and Delays Leaf Senescence Induced by Nitrogen Starvation. Front. Plant. Sci..

[69] Livak K.J., Schmittgen T.D. (2001). Analysis of relative gene expression data using real-time quantitative PCR and the 2(-Delta Delta C(T)) Method. Methods.

[70] Rigaill G., Balzergue S., Brunaud V., Blondet E., Rau A., Rogier O., Caius J., Maugis-Rabusseau C., Soubigou-Taconnat L., Aubourg S. (2016). Synthetic data sets for the identification of key ingredients for RNA-seq differential analysis. Briefings Bioinform..

[71] McCarthy D.J., Chen Y., Smyth G.K. (2012). Differential expression analysis of multifactor RNA-Seq experiments with respect to biological variation. Nucleic Acids Res..

[72] Raudvere U., Kolberg L., Kuzmin I., Arak T., Adler P., Peterson H., Vilo J. (2019). g:Profiler: A web server for functional enrichment analysis and conversions of gene lists (2019 update). Nucleic Acids Res..

[73] Supek F., Bošnjak M., Škunca N., Smuc T. (2011). REVIGO Summarizes and Visualizes Long Lists of Gene Ontology Terms. PLoS ONE.

[74] Barupal D.K., Fiene O. ChemRICH-Chemical Similarity Enrichment Analysis Software. http://chemrich.fiehnlab.ucdavis.edu/.

[75] Barupal D.K., Fiene O. (2017). Chemical Similarity Enrichment Analysis (ChemRICH) as alternative to biochemical pathway mapping for metabolomic datasets. Sci. Rep..

[76] R Core Team (2020). R: A Language and Environment for Statistical Computing.

