# Supplementary material for: Comparative Omics Analysis of Brassica napus Roots Subjected to Six Individual Macronutrient Deprivations Reveals Deficiency-Specific Genes and Metabolomic Profiles
Source: Int J Mol Sci. 2021 Oct 28;22(21):11679. doi: 10.3390/ijms222111679 (PMC8584284; doi:10.3390/ijms222111679)

**Supplementary data SD2**: (A) Nucleotidic sequences of primers for amplification by RT-qPCR of housekeeping (EF1-α and 18s RNA) or target genes used for RNAseq validation. (B) Correlation between log2fold change ratio from RNAseq analysis and $2^{-DDCt}$ of target genes.

**A**

| Gene ID | Primers sequences |
|---|---|
| EF1-α | Forward 5'-TTTCGAGGGTGACAACATGA<br>Reverse 5'-CCGTTCCAATACCACCAATC |
| 18 S | Forward 5'-CGGATAACCGTAGTAATTCTAG<br>Reverse 5'-GTACTCATTCCAATTACCAGAC |
| BnaA03g43750D | Forward 5'-CCAGAAGGTGGTTATGGCTATG<br>Reverse 5'-TGGAGAAGCTGCACCATTTCCA |
| BnaA10g25800D | Forward 5'-CTAATGGACGCATGGTTCTCAC<br>Reverse 5'-GAGATCAAACACATGTGGCTCC |
| BnaA02g35870D | Forward 5'-TTCATAGCGGCTGGTCCTAAAC<br>Reverse 5'-CACTAGCTCTCACGAACACAAC |
| BnaA02g35870D | Forward 5'-TTCATAGCGGCTGGTCCTAAAC<br>Reverse 5'-TTCATAGCGGCTGGTCCTAAAC |
| BnaC02g44160D | Forward 5'-GGTGTTGAAGAGAGTGAAGAGC<br>Reverse 5'-CACTAGCTCTCACGAACACAAC |
| BnaC02g44160D | Forward 5'-GGTGTTGAAGAGAGTGAAGAGC<br>Reverse 5'-ACGTTGTCGTCACTGAAGAGTG |
| BnaC03g56990D | Forward 5'-GTGATATCGGGAATGACAGGAG<br>Reverse 5'-ATTGAGGATGAGTTGTGGCACG |
| BnSultr1.1 | Forward 5'-AGATATTGCGATCGGACCAG<br>Reverse 5'-GAAAACGCCAGCAAAGAAAG |

**B**

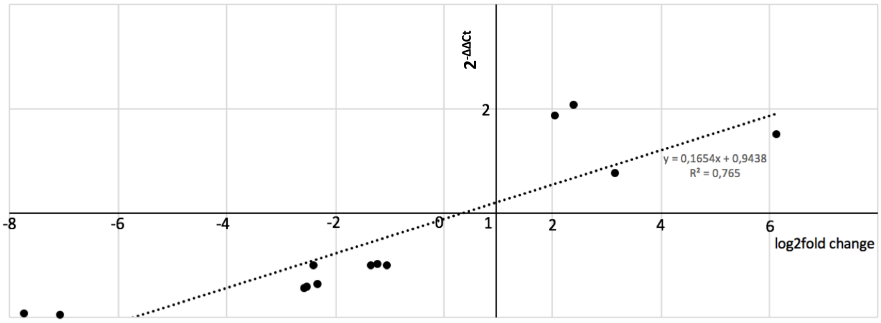

Supplement: Supplementary file 1 [file ijms-22-11679-s001.zip › Supplementary data SD2.pdf]
