# Supplementary material for: Comparative Omics Analysis of Brassica napus Roots Subjected to Six Individual Macronutrient Deprivations Reveals Deficiency-Specific Genes and Metabolomic Profiles
Source: Int J Mol Sci. 2021 Oct 28;22(21):11679. doi: 10.3390/ijms222111679 (PMC8584284; doi:10.3390/ijms222111679)
Supplement: Supplementary file 1 [file ijms-22-11679-s001.zip › Supplementary data SD8.pdf]

Supplementary data SD7: Kinetic of photosynthesis activity of *Brassica napus* subjected to six individual macronutrient deprivations during 9 days.

	Photosythesis (µmol.CO_2.m^{-2}.s^{-1})		
	Day 3	Day 6	Day 9
Control	17.13 ± 1.83	19.2 ± 2.09	18.16 ± 1.47
-N	14.52 ± 1.18	15.23 ± 1.21	6.94* ± 1.29
-Mg	16.09 ± 1.06	19.4 ± 0.81	14.35 ± 2.74
-P	13.62 ± 1.43	16.42 ± 1.52	12.74* ± 1.36
-S	13.47 ± 1.51	20.05 ± 1.39	19.39 ± 1.35
-K	18.44 ± 1.91	20.79 ± 2.07	21.51 ± 1.41
-Ca	15.46 ± 1.09	17.78 ± 2.88	19.86 ± 1.94
