# Supplementary material for: Comparative Omics Analysis of Brassica napus Roots Subjected to Six Individual Macronutrient Deprivations Reveals Deficiency-Specific Genes and Metabolomic Profiles
Source: Int J Mol Sci. 2021 Oct 28;22(21):11679. doi: 10.3390/ijms222111679 (PMC8584284; doi:10.3390/ijms222111679)
Supplement: Supplementary file 1 [file Supplementary data SD1.pdf › Supplementary data SD1.pdf]

Supplementary data SD1: Compositions of nutrient solutions used to submit *Brassica napus* to ten days of N, Mg, P, S, K and Ca individual deprivations as already described by Courbet *et al.,* 2021.

Salts	Salt concentrations (µM) of individual macronutriment deprivated solutions						
	Control	-N	-Mg	-P	-S	-K	-Ca
KNO_3	1000	0	1000	1000	1000	0	3500
$Ca(NO_3)_2 + 4H_2O$	1250	0	1250	1250	1250	1750	0
KH_2PO_4	200	0	200	0	0	0	0
K_2HPO_4	0	200	0	0	200	0	0
$MgSO_4$	400	150	0	400	0	400	400
$MgCl_2$	0	250	0	0	400	0	0
NaFe-EDTA $3H_2O$	50	50	50	50	50	50	50
NaFe-EDDHA	50	50	50	50	50	50	50
Fe-Citrate	0	0	0	0	0	0	0
H_3BO_3	10	10	10	10	10	10	10
$MnSO_4$	3	3	3	3	0	3	3
$MnCl_2$	0	0	0	0	3	0	0
$ZnSO_4$	3	3	3	3	0	3	3
$CuSO_4$	0.7	0.7	0.7	0.7	0	0.7	0.7
$(NH_4)_6Mo_7O_{24}$	0.008	0.008	0.008	0.008	0.008	0.008	0.008
$CoCl_2$	0.1	0.1	0.1	0.1	0.1	0.1	0.1
$NiCl_2$	0.15	0.15	0.15	0.15	0.15	0.15	0.15
$Si(OH)_4$	900	900	900	900	900	900	1100
$CaCl_2 + 2H_2O$	500	750	500	200	0	450	0
KCl	100	400	0	300	100	0	0
Na_2SeO_4	0.01	0.01	0.01	0.01	0.01	0.01	0.01
$CaCO_3$	360	940	0	620	720	0	0
K_2SO_4	100	350	150	100	0	0	100
Na_2SiO_3	200	200	200	200	200	200	0
HCl	600	2220	500	1400	1800	300	900
$CaSO_4$	0	0	350	0	0	100	0
$Ca(H_2PO_4)$	0	0	0	0	0	200	0
$ZnCl_2$	0	0	0	0	3	0	0
$CuCl_2$	0	0	0	0	0.7	0	0
NaCl	0	0	0	0	0	0	200
NaH_2PO_4	0	0	0	0	0	0	200
KOH	0	0	0	0	0	0	1000

Nutrients	Elemental concentrations (µM) of individual macronutriment deprivated solutions						
	Control	-N	-Mg	-P	-S	-K	-Ca
N	3500	0.048	3500	3500	3500	3500	3500
Mg	400	400	0	400	400	400	400
P	200	200	200	0	200	200	200
S	507	507	507	507	0	507	507
K	1500	1500	1500	1500	1500	0	4700
Ca	2110	1690	2100	2070	1970	2500	0
B	10	10	10	10	10	10	10
Cl	2001	4621	1701	2101	2707	1201	1101
Mn	3	3	3	3	3	3	3
Fe	100	100	100	100	100	100	100
Ni	0.15	0.15	0.15	0.15	0.15	0.15	0.15
Cu	0.7	0.7	0.7	0.7	0.7	0.7	0.7
Zn	3	3	3	3	3	3	3
Mo	0.056	0.056	0.056	0.056	0.056	0.056	0.056
Na	500	500	500	500	500	500	500
Al	0	0	0	0	0	0	0
Si	1100	1100	1100	1100	1100	1100	1100
V	0	0	0	0	0	0	0
Co	0.1	0.1	0.1	0.1	0.1	0.1	0.1
Se	0.01	0.01	0.01	0.01	0.01	0.01	0.01
